# Effects of Transcranial Direct Current Stimulation and High-Definition Transcranial Direct Current Stimulation Enhanced Motor Learning on Robotic Transcranial Magnetic Stimulation Motor Maps in Children

**DOI:** 10.3389/fnhum.2021.747840

**Published:** 2021-10-06

**Authors:** Adrianna Giuffre, Ephrem Zewdie, James G. Wrightson, Lauran Cole, Helen L. Carlson, Hsing-Ching Kuo, Ali Babwani, Adam Kirton

**Affiliations:** ^1^Calgary Pediatric Stroke Program, Alberta Children’s Hospital, Calgary, AB, Canada; ^2^Department of Pediatrics, Cumming School of Medicine, University of Calgary, Calgary, AB, Canada; ^3^Department of Clinical Neurosciences, Cumming School of Medicine, University of Calgary, Calgary, AB, Canada; ^4^Department of Physical Medicine & Rehabilitation, University of California, Davis, Sacramento, CA, United States

**Keywords:** robotic TMS, motor mapping, tDCS and HD-tDCS, motor learning, pediatrics, neurophysiology, neuroplasticity

## Abstract

**Introduction:** Conventional transcranial direct current stimulation (tDCS) and high-definition tDCS (HD-tDCS) may improve motor learning in children. Mechanisms are not understood. Neuronavigated robotic transcranial magnetic stimulation (TMS) can produce individualised maps of primary motor cortex (M1) topography. We aimed to determine the effects of tDCS- and HD-tDCS-enhanced motor learning on motor maps.

**Methods:** Typically developing children aged 12–18 years were randomised to right M1 anodal tDCS, HD-tDCS, or Sham during training of their left-hand on the Purdue Pegboard Task (PPT) over 5 days. Bilateral motor mapping was performed at baseline (pre), day 5 (post), and 6-weeks retention time (RT). Primary muscle was the first dorsal interosseous (FDI) with secondary muscles of abductor pollicis brevis (APB) and adductor digiti minimi (ADM). Primary mapping outcomes were volume (mm^2^/mV) and area (mm^2^). Secondary outcomes were centre of gravity (COG, mm) and hotspot magnitude (mV). Linear mixed-effects modelling was employed to investigate effects of *time and stimulation type (tDCS, HD-tDCS, Sham)* on motor map characteristics.

**Results:** Twenty-four right-handed participants (median age 15.5 years, 52% female) completed the study with no serious adverse events or dropouts. Quality maps could not be obtained in two participants. No effect of time or group were observed on map area or volume. LFDI COG (mm) differed in the medial-lateral plane (x-axis) between tDCS and Sham (p = 0.038) from pre-to-post mapping sessions. Shifts in map COG were also observed for secondary left-hand muscles. Map metrics did not correlate with behavioural changes.

**Conclusion:** Robotic TMS mapping can safely assess motor cortex neurophysiology in children undergoing motor learning and neuromodulation interventions. Large effects on map area and volume were not observed while changes in COG may occur. Larger controlled studies are required to understand the role of motor maps in interventional neuroplasticity in children.

## Introduction

Transcranial direct current stimulation (tDCS) is a non-invasive technique capable of modulating cortical excitability in humans (Nitsche and Paulus, 2000). During conventional tDCS, a sub-threshold current is applied to the brain via two sponge electrodes placed on the scalp. Prolonged enhancement of primary motor cortex (M1) excitability following anodal tDCS has been evidenced by an increase in motor evoked potential (MEP) amplitude within hand muscles (Nitsche and Paulus, 2000). Anodal tDCS applied simultaneously with behavioural tasks facilitates enhanced motor performance over a single session (Nitsche et al., 2003; Boggio et al., 2006; Vines et al., 2006) or across multiple sessions in healthy adults (Reis et al., 2009) with effects outlasting the stimulation period (Nitsche and Paulus, 2001; Boggio et al., 2006; Reis et al., 2009; Matsuo et al., 2011; Sohn et al., 2012; Kidgell et al., 2013). tDCS shows promise for facilitating motor recovery post stroke (for review see, Kang et al., 2016) and a range of other neurological disorders (Lefaucheur, 2016). High-definition tDCS (HD-tDCS) offers improved focal targeting of cortical areas and stronger regional electric fields by centering four electrodes around a central electrode of opposite polarity in a 4 × 1 ring-like orientation (Datta et al., 2009; Dmochowski et al., 2011; Villamar et al., 2013; Alam et al., 2016). Behavioural improvements occur in healthy adults over single (Doppelmayr et al., 2016) and multiple sessions (Pixa et al., 2017) in which patterns of cortical excitability may outlast those induced by conventional tDCS (Kuo et al., 2013).

Children have been neglected in neuromodulation research. With a well-defined safety and tolerability profile (Bikson et al., 2016) in adults and clear clinical need, early translation towards tDCS applications in pediatric populations seem favourable. However, limited evidence suggests primary principles of tDCS, such as polarity and current strength, may differ in the developing brain (Moliadze et al., 2015). Preliminary investigations in pediatric populations highlight potentially varying effects of tDCS in children (Kessler et al., 2013; Rajapakse and Kirton, 2013; Bikson et al., 2016). Our team recently demonstrated that M1-tDCS over three consecutive days enhances motor learning in healthy children with effects retained at 6-weeks (Ciechanski and Kirton, 2017). We have also demonstrated that both anodal tDCS and HD-tDCS can enhance motor learning over multiple days with retained effects (Cole et al., 2018). However, the underlying mechanisms of such interventional neuroplasticity remain unknown.

Transcranial magnetic stimulation (TMS) can produce maps of cortical representations of individual muscles within M1 (Cohen and Hallett, 1988; Wassermann et al., 1992; Wilson et al., 1993; Pascual-Leone et al., 1995b; Thickbroom et al., 1998) and is increasingly applied for clinical applications such as preoperative neurosurgical planning for brain tumor removal and epilepsy surgery (Picht et al., 2011; Lefaucheur and Picht, 2016), for review see Sollmann et al. (2021). Common characteristics, such as map volume, area, hotspot magnitude, and centre of gravity (COG), may also potentially quantify changes in cortical neurophysiology following learning or stimulation-induced changes in motor performance (Cohen et al., 1993; Pascual-Leone et al., 1995a, b). For instance, an increase in motor map size following the acquisition of new fine motor skills in healthy adults (Pascual-Leone et al., 1995a) resembles cortical muscle rearrangement following behavioural motor training in primates (Donoghue et al., 1992; Recanzone et al., 1992; Nudo et al., 1996). TMS motor maps, including robotic neuronavigated methods, have also been acquired in the developing brain of healthy and clinical populations (Maegaki et al., 1999; Garvey et al., 2003; Garvey and Gilbert, 2004; Friel et al., 2017; Grab et al., 2018; Giuffre et al., 2019b, 2021). Whether such advanced mapping methods can detect interventional changes in motor cortex neurophysiology during modulated motor learning in children has not been studied. Here, we aimed to determine the potential effects of tDCS and HD-tDCS enhanced motor learning on robotic TMS motor maps. Based on preliminary evidence from adult studies (Pascual-Leone et al., 1995a; Classen et al., 1998), we hypothesised that map volume and area of the trained first dorsal interosseous muscle (FDI) would increase following active stimulation (tDCS and HD-tDCS) and be associated with enhanced motor performance. We also investigated the effects of stimulation and tDCS-enhanced motor performance on additional motor map outcomes, hotspot magnitude (MEP amplitude), and COG of both hands.

## Materials and Methods

### Participants and Study Design

Participants were recruited from the community including a population-based research cohort, the Healthy Infants and Children Clinical Research Program (HICCUP). Inclusion criteria were (1) typical neurodevelopment, (2) 12–18 years of age, (3) right-handed (Edinburgh handedness inventory with a laterality index ≥28 (Oldfield, 1971), (4) informed assent/consent, and (5) no contraindications to MRI, TMS, or tDCS (Keel et al., 2001). Children with neurodevelopmental or neuropsychiatric diagnosis or taking neuropsychiatric medications were excluded.

**FIGURE 1:**
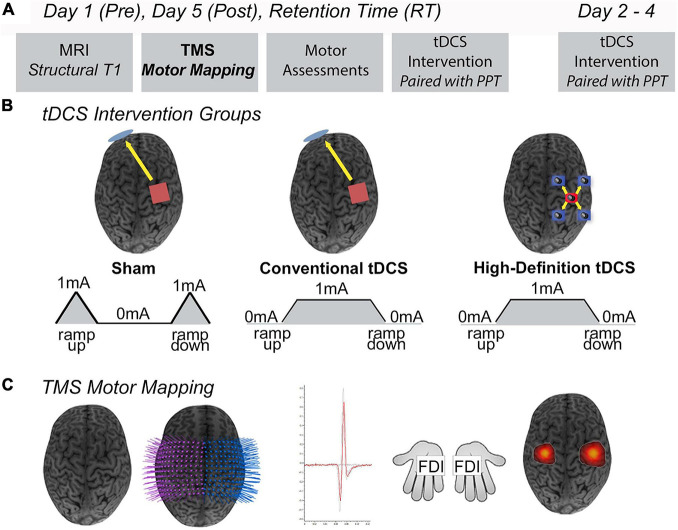
Study design. **(A)** On Day 1 (pre), participants completed an MRI protocol (90 min), robotic TMS motor mapping (90 min), and the Purdue Pegboard Task (PPT) (10 min), with a 15-min break between each assessment. Days 2–4, participants received five consecutive days of tDCS while training their left hand on the PPT task (PPT_L_). Day 5 (post), participants received their final tDCS stimulation protocol and proceeded to complete the same MRI protocol, TMS motor mapping, and PPT as Day 1. At a 6-weeks retention time (RT), participants repeated all assessments received on Day 1 (MRI, robotic TMS motor mapping, PPT). **(B)** *Intervention groups.* Anodal conventional 1 × 1 Sham tDCS, conventional 1 × 1 tDCS, or 4 × 1 High-Definition tDCS (HD-tDCS) was applied for 20 min at 1 mA targeting the right primary motor cortex (M1). Shown are positions of anode electrodes (red), cathode electrode(s) (blue), direction of current flow (yellow arrows). **(C)** *Robotic TMS motor mapping.* Three-dimensional (3D) curvilinear brains were reconstructed from participants T1-weighted anatomical images. Bilateral 12 × 12 grids (7 mm) spacing were centered over the hand knob of right M1 (stimulated cortex) and left M1. Average motor evoked potential (MEP) amplitudes (mV) were acquired from the FDI (target muscle) and other hand muscles. Visualization of robotic TMS motor maps of the FDI muscle overlaid on 3D curvilinear brain.

Participants and/or their guardian consented/assented to participate in the Accelerated Motor Learning in Pediatrics (AMPED) randomised, double-blind interventional trial (NCT03193580) (Figure 1). Details of the protocol are described in detail elsewhere (Cole et al., 2018). Briefly, the current study investigated the effects of conventional tDCS and HD-tDCS enhanced motor learning on robotic TMS motor maps in healthy children. At baseline (pre), participants received an MRI (90 min), bilateral robotic TMS motor mapping (90 min), and completed a motor assessment (10 min), with a 15-min break between each assessment, all of which are described below. Participants were then computer randomised to right M1, anodal (1) tDCS, (2) HD-tDCS, or (3) Sham during training of their left-hand on the Purdue Pegboard Task (PPT) over five consecutive days (Figure 1B). All outcome measurements were repeated on day 5 (post) and at a retention time (RT) of 6-weeks.

### Magnetic Resonance Imaging

Images were obtained at the ACH Diagnostic Imaging Centre using a 3T General Electric MR750w scanner (GE Healthcare, Chicago, IL, United States) with a 32-channel head coil using a fast-spoiled gradient echo sequence (FSPGR BRAVO, 226 axial slices, TR = 8.5 ms, TE = 3.2 ms, voxels = 1 mm isotropic). Anatomical high-resolution T1 sequences were transferred to the neuronavigation software (Brainsight2, Rogue, Montreal).

### Robotic TMS Motor Mapping

Prior to the onset of the stimulation intervention, robotic TMS was used to locate the left-hand FDI (LFDI) hotspot. A figure-of-eight 70mm Air-Film coil (Magstim, Dyfed, United Kingdom) was applied to the scalp over the left hand-knob area (right M1) (Yousry et al., 1997). The LFDI hotspot (described below) was localised using single pulse robotic TMS (Axilum Robotics, Strasbourg, France) via a motor mapping technique (Giuffre et al., 2019b), and marked with a Sharpie (permanent marker), serving as the target site for the anode. For the tDCS and Sham groups, two saline-soaked sponge electrodes (25 cm^2^, SNAPpad, Soterix Medical Inc., NY, United States) were applied to the scalp. As described, the anode was placed over the LFDI hotspot, while the cathode was placed over the contralateral supraorbital area. Electrodes were held in place using a plastic headband (SNAPstrap, Soterix Medical Inc., NY, United States). In the HD-tDCS, participants wore an EEG cap with the anode electrode centered over the LFDI hotspot, and four surrounding cathode electrodes positioned in a ring-like orientation (1 cm diameter circular electrodes, electrode holder and gel; Soterix Medical Inc., NY, United States).

Bilateral motor maps were acquired using neuronavigated robotic TMS (Axilum Robotics, Strasbourg, France). The detailed robotic motor mapping protocol can be found elsewhere (Giuffre et al., 2019b). Anatomical T1-images were co-registered with each participant using an optical detection camera system (Polaris, NDI Medical Solutions, Waterloo, ON, Canada) and used to reconstruct skin and curvilinear brain. A 12 × 12 rectangular grid with 7 mm spacing was superimposed on the reconstructed curvilinear brain and centered over the anatomical hand-knob of left and right M1 (Yousry et al., 1997) to generate targets for motor mapping. Each grid-point trajectory was aligned tangentially to the cortical surface and maintained at 45° in relation to the interhemispheric fissure using a figure-of-eight 70 mm Air-Film coil (Magstim, Dyfed, United Kingdom), accurately maintaining position and motion correction in near real-time (1 cm/s) (Ginhoux et al., 2013; Goetz et al., 2019).

Participants were seated in a comfortable chair with both arms resting and given an option to watch a movie. Ag-AgCl electrodes (Kendall, Chicopee, MA, United States) were placed on both hands over three muscles: FDI, abductor pollicis brevis (APB), and adductor digiti minimi (ADM). MEP were captured using surface electromyography (EMG), amplified (gain = 1,000, Bortec Biomedical, Calgary, AB, Canada) and filtered (20–2,500 Hz) using a CED 1401 signal analog/digital converter (Cambridge Electronic Design Limited, Cambridge, United Kingdom), and digitised at a rate 5,000 Hz (Signal 6.0 software, Cambridge Electronic Design Limited, Cambridge, United Kingdom).

Experiments began by mapping the right-hemisphere. The LFDI hotspot was first determined as the largest, most consistent MEP (mV). In addition, the FDI hotspot of each hand was used to determine the resting motor threshold (RMT) and mapping intensity. RMT was extrapolated from 5% of the slope of a stimulus response curve (SRC) (Ridding and Rothwell, 1997; Temesi et al., 2014). RMT was used to determine the mapping intensity (120% RMT) for each session. Mapping was performed at a machine stimulator output (MSO) intensity of 120% RMT (Ridding and Rothwell, 1997, 2007) or 100% MSO if their RMT were too high (>84% MSO).

Beginning at the FDI hotspot, four single TMS pulses at 1-second inter-stimulus intervals (1 Hz) were delivered to each grid-point. A grid-point was deemed responsive if ≥2/4 MEP had peak-to-peak amplitudes ≥50 μV in at least one of the three hand-muscles. The neuronavigated robotic system moved to each successive grid-point until a non-responsive grid-point was reached, generating the first border of the map. Motor mapping was completed once non-responsive grid-points formed a complete perimeter in each hemisphere. The complete TMS motor mapping protocol and representative three-dimensional (3D) motor maps of the LFDI muscles (target muscle) are shown in Figure 1C.

Bilateral robotic TMS motor maps were then analysed using a custom mapping script (MATLAB R2016b, The MathWorks, Inc., Natick, MA, United States). The LFDI was the primary muscle as it served as the cortical target site for the tDCS intervention. Primary (map volume and area) and secondary map outcomes were obtained for both hands and characterised by the following:

1. Volume (mm^2^/mV): Averaged peak-to-peak MEP amplitude at each responsive grid-point multiplied by the summated active grid area (mm^2^).
2. Area (mm^2^): Binarised MEP amplitudes of positive number of responsive grid-points (average ≥ 2/4 MEP amplitudes ≥50 μV) multiplied by grid area (7 mm × 7 mm = 49mm^2^).
3. Centre of Gravity (COG) (mm): Coordinates of the map centroid calculated by using the weighted distribution of the largest MEP amplitude (Wassermann et al., 1992), in a 2 dimensional (2D) x–y plane, assuming z is equal to zero at the surface of the head. COG in the medial-lateral plane corresponds to the 2D x-axis (COG-x), while the anterior-posterior plane corresponds to the 2D y-axis (COG-y). Xi and yi are the respective x-, y- coordinates of the location where the peak-to-peak MEP amplitude (Mi) was recorded. $xCOG=\frac{\sum xiMi}{\sum Mi}yCOG=\frac{\sum yiMi}{\sum Mi}$

### Safety and Tolerability

Immediately following each robotic TMS mapping session, participants completed a pediatric non-invasive brain stimulation safety and tolerability questionnaire (Zewdie et al., 2020). The safety and tolerability of tDCS and HD-tDCS sessions are reported elsewhere (Cole et al., 2018; Zewdie et al., 2020). The mapping experience was ranked against common childhood experiences; (1) play a game, (2) birthday party, (3) watch TV, (4) long car ride, (5) go to dentist, (6) shot at the doctor, and (7) throwing-up. Participants were also screened for symptoms of headache, neck pain, unpleasant tingling, light-headedness, nausea, and any other self-reported symptoms, all of which were graded as mild, moderate, or severe.

### Conventional and High Definition tDCS and Trained Motor Task

At baseline, participants performed the PPT, a validated measure of hand dexterity, using their left-hand (PPT_*L*_) and right-hand (PPT_*R*_) (Gardner and Broman, 1979). During the tDCS intervention, participants received anodal stimulation targeting the right M1 while training their left-hand on the PPT (Figure 1B). Over five consecutive days, participants performed the PPT_*L*_ at minutes 5, 10, and 15, while receiving one of the three stimulation interventions, and once again after the stimulation period ended. At post and RT, participants repeated the PPT (PPT_*L*_ and PPT_*R*_) assessment to examine the effects of tDCS and HD-tDCS on motor learning and retention.

### Statistical Analysis

Analyses were performed using the R statistical software package (RStudio Team, 2015) using jamovi (Version 1.6, Sydney, Australia^1^). Data were reported as mean and standard error (SE) unless otherwise stated. Robotic TMS motor map outcomes and motor assessments were tested for normality using the Shapiro–Wilk test. Repeated measures analysis of variance (ANOVA) determined potential differences of RMT across time by group. One-way ANOVA or Kruskal-Wallis H Test determined potential differences of muscles and outcomes at baseline across intervention groups. Linear mixed-effects modeling with restricted maximum likelihood estimation was used to investigate potential effects of *time* (pre, post, and RT) and *group*, controlling for potential effects of age and sex. We collapsed active tDCS and HD-tDCS into one stimulation group (*stimulation*) to investigate the effects of active stimulation on motor maps. Subsequently, we modeled each type of tDCS (tDCS, HD-tDCS, and Sham), *stimulation type*, separately to explore whether specific stimulation montages might alter motor maps. Dependent variables included primary outcomes (map volume and area) and secondary outcomes (hotspot magnitude and COG). Post-hoc analyses were corrected for multiple comparisons (Holm-Sidak). Estimated marginal means and fixed parameter effects are reported for the left-hand FDI (target muscle) for primary and secondary motor map outcomes. Lastly, linear regression explored the association between change in FDI map volume and change in PPT motor performance from pre-to-post mapping sessions. Estimated marginal means of motor map outcomes for left-hand secondary muscles and right-hand muscles are reported in the Supplementary Material.

## Results

### Participants

Twenty-four participants were recruited and completed the AMPED trial (12–18 years old, median age 15.5 years, 13 female). Two participants at baseline and one participant at RT received unilateral motor mapping of the right-hemisphere (stimulated cortex) only due to time constraints. One participant was excluded from the motor mapping analyses as their motor mapping intensity exceeded 100% MSO, resulting in insufficient MEP recordings. An additional participant was excluded as MEP could not be recorded from the FDI muscle. Demographics and baseline PPT scores across groups are reported in Table 1. The final sample included 22 right-hemisphere (stimulated) motor maps (*n* = 7 Sham, *n* = 8 tDCS, *n* = 7 HD-tDCS) and 20 left-hemisphere (non-stimulated) motor maps (*n* = 7 Sham, *n* = 8 tDCS, *n* = 5 HD-tDCS). The primary outcomes were not associated with age or sex and the following results held true when controlling for age and sex across motor map outcomes.

**TABLE 1:** Demographics and mean (SD) baseline PPT scores.

**Group**	***N***	**Age**	**Sex (F:M)**	**Baseline PPT_*L*_**	**Baseline PPT_*R*_**
Sham	7	15.61 (1.31)	2:5	12.52 (0.84)	14.90 (1.89)
tDCS	8	15.94 (1.54)	6:2	13.50 (1.32)	15.21 (1.90)
HD-tDCS	7	15.12 (1.91)	3:4	14.10 (1.82)	15.90 (1.63)
Mean	22	15.58 (1.56)	11:11	13.38 (1.47)	15.33 (1.78)

*Age, age in years at enrollment; Baseline PPT_*L*_, left-hand PPT scores at baseline (number of pegs); Baseline PPT_*R*_, right-hand PPT scores at baseline (number of pegs); PPT, Purdue Pegboard Task; SD, standard deviation.*

### Effects of tDCS and HD-tDCS on Motor Learning

The effects of stimulation on motor learning are described in detail elsewhere (Cole et al., 2018). Briefly, on the primary trained motor task (PPT_*L*_) all participants showed an increase in number of pegs placed over five consecutive days of training, regardless of tDCS intervention (*p* < 0.001). Participants in the active stimulation groups (tDCS and HD-tDCS) had significantly enhanced rates of motor learning compared to Sham [tDCS *t*_(117)_ = 2.058, *p* = 0.042; HD-tDCS *t*_(117)_ = 1.986, *p* = 0.049]. Effects were sustained at 6 weeks retention time.

### Mapping and Thresholds

**FIGURE 2:**
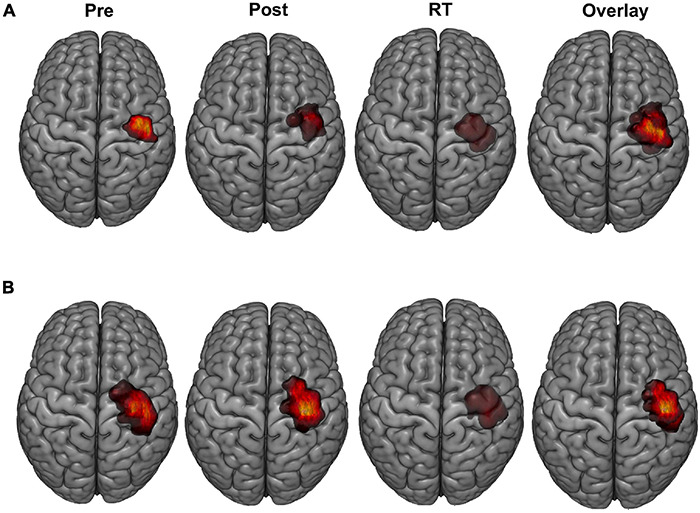
LFDI motor maps. Representative single subject examples of LFDI motor map across robotic TMS motor mapping sessions (pre, post, RT) and all three time points overlaid on a three-dimensional (3D) curvilinear brain from a Sham participant **(A)** and a participant in the tDCS group **(B)**. Pre = baseline; Post = day 5; RT = six-weeks retention time; LFDI = left-hand first dorsal interosseous muscle.

Representative examples of LFDI motor maps overlaid on a 3D anatomical brain from one Sham participant and one tDCS participant across mapping sessions are depicted in Figure 2. Bilateral motor mapping was performed with a mean time of 37 min (SD 12 min, ranging 9–31 min) and was comparable between hemispheres.

Resting motor threshold was similar across treatment groups, mapping sessions and between the LFDI (pre = 60%, post = 58%, RT = 59%) and right-hand first dorsal interosseous (RFDI) (pre = 59%, post = 58%, RT = 58%). Linear mixed-effects modeling demonstrated no effects of *time, stimulation*, or interaction between *time* and *stimulation* on RMT in either FDI.

### Motor Map Primary Outcomes in the LFDI Muscle

**FIGURE 3:**
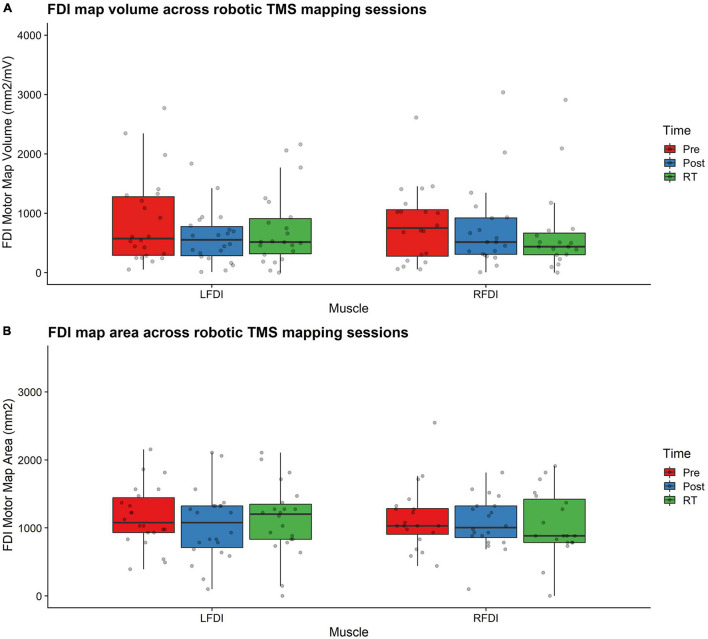
FDI map volume and area. **(A)** FDI map volume (mm^2^/mV) and **(B)** area (mm^2^) across robotic TMS motor mapping sessions. Map volume (mm^2^/mV) = Cumulative active map area × MEP amplitude (mV) at each responsive grid-point; Map area (mm^2^) = Grid spacing (49mm^2^) × number of responsive grid-points; LFDI = left-hand first dorsal interosseous muscle; RFDI = right-hand first dorsal interosseous muscle; Pre = baseline (red); Post = day 5 (blue); RT = six-weeks retention time (green). Box ends are interquartile range, the line is median, and the whiskers are 5–95%.

Bilateral FDI motor map outcomes of volume and area across mapping sessions are shown in Figure 3 and described in detail below for the trained LFDI.

#### LFDI Map Volume

**FIGURE 4:**
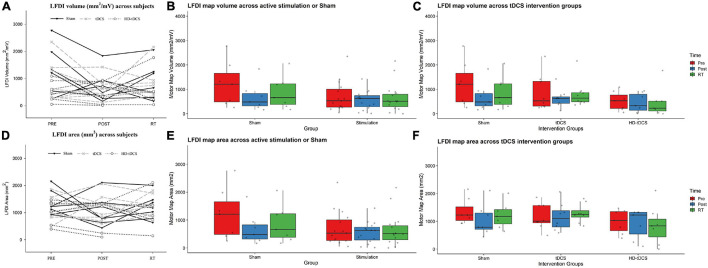
LFDI map volume and area across robotic TMS motor mapping sessions. **(A)** LFDI map volume (mm^2^/mV) of each participant across mapping sessions. **(B)** LFDI map volume (mm^2^/mV) in active stimulation groups (tDCS and HD-tDCS) and Sham. **(C)** LFDI map volume (mm^2^/mV) in intervention groups (tDCS, HD-tDCS and Sham). **(D)** LFDI map area (mm^2^) of each participant across motor mapping sessions. **(E)** LFDI map area (mm^2^) in active stimulation groups (tDCS and HD-tDCS) and Sham. **(F)** LFDI map area (mm^2^) in intervention groups (tDCS, HD-tDCS, and Sham). Map volume (mm^2^/mV) = Cumulative active map area × MEP amplitude (mV) at each responsive grid-point; Map area (mm^2^) = Grid spacing (49mm^2^) × number of responsive grid-points; Black line with triangle = Sham participants; Gray dashed line with X = tDCS participants; Gray dotted line with circle = HD-tDCS; LFDI = left-hand first dorsal interosseous muscle; Pre = baseline; Post = day 5; RT = six-weeks retention time. Box ends are interquartile range, the line is median, and the whiskers are 5–95%.

Left-hand FDI map volume and area across participants, stimulation (Active vs Sham) and stimulation type (tDCS, HD-tDCS, and Sham), and mapping sessions are depicted in Figure 4. Estimated marginal means of LFDI map volume are reported in Table 2. LFDI map volumes appeared stable across sessions (Figure 4A). No effects of *time* (*F* = 3.12, *p* = 0.055), *stimulation* (*F* = 1.34, *p* = 0.268), or any interaction between *time* and *stimulation* (*F* = 1.01, *p* = 0.373) were observed on LFDI map volume between stimulation groups (Figure 4B). LFDI map volumes were comparable at the pre timepoint between stimulation groups (*F* = 1.642, *p* = 0.220). There were no effects of *time* (*F* = 2.23, *p* = 0.122), *stimulation type* (*F* = 0.91, *p* = 0.431), or any interaction between *time* and *stimulation type* (*F* = 0.57, *p* = 0.684) on LFDI motor map volume when examined across the three intervention groups (Figure 4C). Fixed parameter effects of LFDI volume across both any stimulation and each stimulation type are reported in Tables 3, 4.

**TABLE 2:** LFDI estimated marginal means (SE) of robotic TMS motor map outcomes across intervention groups.

**Estimated Marginal Means Intervention Groups (tDCS, HD-tDCS, Sham)**															
	**Time**			**Group**			**Time × Group**								
	**PRE**	**POST**	**RT**	**tDCS**	**HD-tDCS**	**Sham**	**tDCS**			**HD-tDCS**			**Sham**		
							**PRE**	**POST**	**RT**	**PRE**	**POST**	**RT**	**PRE**	**POST**	**RT**
LFDI Volume (mm^2^/mV)	867 (130)	589 (130)	717 (130)	773 (174)	476 (186)	923 (186)	869 (215)	635 (215)	846 (215)	518 (230)	448 (230)	464 (230)	1216 (230)	683 (230)	871 (230)
LFDI Area (mm^2^)	1165 (107)	1039 (107)	1117 (107)	1203 (148)	917 (148)	1202 (148)	1164 (177)	1158 (177)	1286 (177)	987 (189)	910 (189)	854 (189)	1344 (189)	1050 (189)	1211 (189)
LFDI COG-x (mm)	6.59 (0.151)	6.69 (0.151)	6.77 (0.151)	6.93 (0.225)	6.41 (0.241)	6.70 (0.241)	6.63 (0.249)	7.21 (0.249)	6.97 (0.249)	6.32 (0.267)	6.34 (0.267)	6.56 (0.267)	6.81 (0.267)	6.51 (0.267)	6.78 (0.267)
LFDI COG-y (mm)	5.40 (0.146)	5.55 (0.146)	5.51 (0.146)	5.38 (0.200)	5.78 (0.214)	5.30 (0.214)	5.21 (0.242)	5.37 (0.242)	5.57 (0.242)	5.63 (0.259)	5.79 (0.259)	5.93 (0.259)	5.36 (0.259)	5.49 (0.259)	5.05 (0.259)
LFDI Hotspot magnitude (mV)	2.03 (0.341)	1.67 (0.341)	1.65 (0.341)	1.87 (0.508)	1.14 (0.543)	2.34 (0.543)	2.02 (0.564)	1.81 (0.564)	1.79 (0.564)	1.25 (0.603)	1.15 (0.603)	1.02 (0.603)	2.80 (0.603)	2.06 (0.603)	2.15 (0.603)

*LFDI, left-hand first dorsal interosseous muscle; SE, standard error; COG, centre of gravity; COG-x, medial-lateral plane; COG-y, anterior-posterior plane; Hotspot magnitude, MEP amplitude; Pre, baseline; Post, day 5; RT, six-weeks retention time.*

**TABLE 3:** LFDI fixed parameters for motor map outcomes across Active and Sham stimulation (stimulation).

	**Fixed Parameter effects stimulation type (Active tDCS vs Sham)**						
**LFDI**	**Names**	**Effect**	**Estimate**	**SE**	**df**	***t***	***p***
Volume (mm^2^/mV)	(Intercept)	(Intercept)	779	113	20.0	6.860	<0.001
	Time1	Post − Pre	−345	139	40.0	–2.490	**0.017**
	Time2	RT − Pre	−199	139	40.0	–1.440	0.159
	Group	Stimulation − Sham	−288	227	20.0	–1.270	0.219
	Time1 × Group	Post − Pre × Stimulation − Sham	376	277	40.0	1.350	0.183
	Time2 × Group	RT − Pre × Stimulation − Sham	291	277	40.0	1.050	0.300
Area (mm^2^)	(Intercept)	(Intercept)	1136	92	20.0	12.369	<0.001
	Time1	Post − Pre	−167	122	40.0	–1.365	0.180
	Time2	RT − Pre	−65	122	40.0	–0.532	0.598
	Group1	Stimulation − Sham	−132	184	20.0	–0.721	0.479
	Time1 × Group1	Post − Pre × Stimulation − Sham	255	244	40.0	1.044	0.303
	Time2 × Group1	RT − Pre × Stimulation − Sham	136	244	40.0	0.558	0.580
COG x-axis (mm)	(Intercept)	(Intercept)	6.695	0.151	20.0	44.266	<0.001
	Time1	Post − Pre	0.008	0.124	40.0	0.068	0.946
	Time2	RT − Pre	0.133	0.124	40.0	1.065	0.293
	Group1	Stimulation − Sham	−0.013	0.302	20.0	–0.042	0.967
	Time1 × Group1	Post − Pre × Stimulation − Sham	0.619	0.249	40.0	2.488	**0.017**
	Time2 × Group1	RT − Pre × Stimulation − Sham	0.315	0.249	40.0	1.264	0.213
COG y-axis (mm)	(Intercept)	(Intercept)	5.435	0.132	20.0	41.122	<0.001
	Time1	Post − Pre	0.143	0.149	40.0	0.960	0.343
	Time2	RT − Pre	0.007	0.149	40.0	0.050	0.961
	Group1	Stimulation − Sham	0.268	0.264	20.0	1.015	0.322
	Time1 × Group1	Post − Pre × Stimulation − Sham	0.031	0.297	40.0	0.106	0.916
	Time2 × Group1	RT − Pre × Stimulation − Sham	0.648	0.297	40.0	2.180	**0.035**
Hotspot Magnitude (mV)	(Intercept)	(Intercept)	1.934	0.328	20.0	5.888	<0.001
	Time1	Post − Pre	−0.453	0.268	40.0	–1.690	0.099
	Time2	RT − Pre	−0.443	0.268	40.0	–1.654	0.106
	Group1	Stimulation − Sham	−0.808	0.657	20.0	–1.229	0.233
	Time1 × Group1	Post − Pre × Stimulation − Sham	0.573	0.536	40.0	1.070	0.291
	Time2 × Group1	RT − Pre × Stimulation − Sham	0.418	0.536	40.0	0.780	0.440

*LFDI, left-hand first dorsal interosseous muscle; COG, centre of gravity; COG-x, medial-lateral plane; COG-y, anterior-posterior plane; Hotspot magnitude, MEP amplitude; df, degrees of freedom; *t*, *t*-statistic; p, significance value (*p* < 0.05 = significant). Bold values represent statistically significant results (*p* < 0.05).*

**TABLE 4:** LFDI fixed parameters for motor map outcomes across intervention groups (stimulation type).

	**Fixed Parameter effects stimulation type (tDCS, HD-tDCS, Sham)**						
**LFDI**	**Names**	**Effect**	**Estimate**	**SE**	**df**	**t**	**p**
Volume (mm^2^/mV)	(Intercept)	(Intercept)	724	105	19.0	6.900	<0.001
	Time1	Post − Pre	−279	132	38.0	–2.109	**0.042**
	Time2	RT − Pre	−151	132	38.0	–1.140	0.261
	Group1	tDCS − Sham	−149	254	19.0	–0.588	0.564
	Group2	HD-tDCS − Sham	−446	263	19.0	–1.700	0.105
	Time1 × Group1	Post − Pre × tDCS − Sham	299	320	38.0	0.933	0.356
	Time2 × Group1	RT − Pre × tDCS − Sham	292	320	38.0	0.911	0.368
	Time1 × Group2	Post − Pre × HD-tDCS − Sham	464	331	38.0	1.401	0.169
	Time2 × Group2	RT − Pre × HD-tDCS − Sham	291	331	38.0	0.879	0.385
Area (mm^2^)	(Intercept)	(Intercept)	1107	84	19.0	13.235	<0.001
	Time1	Post − Pre	−126	116	38.0	–1.088	0.283
	Time2	RT − Pre	−48	116	38.0	–0.414	0.681
	Group1	tDCS − Sham	1	203	19.0	0.004	0.997
	Group2	HD-tDCS − Sham	−285	209	19.0	–1.360	0.190
	Time1 × Group1	Post − Pre × tDCS − Sham	288	280	38.0	1.029	0.310
	Time2 × Group1	RT − Pre × tDCS − Sham	256	280	38.0	0.913	0.367
	Time1 × Group2	Post − Pre × HD-tDCS − Sham	217	289	38.0	0.751	0.457
	Time2 × Group2	RT − Pre × HD-tDCS − Sham	−2.10e−12	289	38.0	−7.25*e*−15	1.000
COG-x (mm)	(Intercept)	(Intercept)	6.681	0.136	19.0	49.105	<0.001
	Time1	Post − Pre	0.232	0.330	19.0	0.704	0.490
	Time2	RT − Pre	-0.292	0.340	19.0	–0.859	0.401
	Group1	tDCS − Sham	0.099	0.112	38.0	0.884	0.382
	Group2	HD−tDCS − Sham	0.183	0.112	38.0	1.625	0.112
	Time1 × Group1	Post − Pre × tDCS − Sham	0.878	0.272	38.0	3.225	**0.003**
	Time2 × Group1	RT − Pre × tDCS − Sham	0.323	0.281	38.0	1.150	0.258
	Time1 × Group2	Post − Pre × HD-tDCS − Sham	0.362	0.272	38.0	1.329	0.192
	Time2 × Group2	RT − Pre × HD-tDCS − Sham	0.261	0.281	38.0	0.927	0.360
COG-y (mm)	(Intercept)	(Intercept)	5.488	0.121	19.0	45.424	<0.001
	Time1	Post − Pre	0.148	0.142	38.0	1.040	0.305
	Time2	RT − Pre	0.114	0.142	38.0	0.801	0.428
	Group1	tDCS − Sham	0.083	0.293	19.0	0.282	0.781
	Group2	HD−tDCS − Sham	0.481	0.302	19.0	1.590	0.128
	Time1 × Group1	Post − Pre × tDCS − Sham	0.032	0.345	38.0	0.094	0.926
	Time2 × Group1	RT − Pre × tDCS − Sham	0.6801	0.345	38.0	1.973	0.056
	Time1 × Group2	Post − Pre × HD-tDCS − Sham	0.0304	0.356	38.0	0.085	0.932
	Time2 × Group2	RT − Pre × HD-tDCS − Sham	0.6120	0.356	38.0	1.719	0.094
Hotspot Magnitude (mV)	(Intercept)	(Intercept)	1.783	0.307	19.0	5.811	<0.001
	Time1	Post − Pre	−0.355	0.256	38.0	–1.134	0.175
	Time2	RT − Pre	−0.373	0.256	38.0	–1.456	0.153
	Group1	tDCS − Sham	−0.466	0.743	19.0	–0.627	0.538
	Group2	HD-tDCS − Sham	−1.198	0.768	19.0	–0.1560	0.135
	Time1 × Group1	Post − Pre × tDCS − Sham	.0524	0.621	38.0	0.643	0.404
	Time2 × Group1	RT − Pre × tDCS − Sham	0.418	0.621	38.0	0.672	0.505
	Time1 × Group2	Post − Pre × HD-tDCS − Sham	0.630	0.641	38.0	0.983	0.332
	Time2 × Group2	RT − Pre × HD-tDCS − Sham	0.418	0.641	38.0	0.651	0.519

*LFDI, left-hand first dorsal interosseous muscle; COG, centre of gravity; COG-x, medial-lateral plane; COG-y, anterior-posterior plane; Hotspot magnitude, MEP amplitude; df, degrees of freedom; *t*, *t*-statistic; *p*, significance value (*p* < 0.05 = significant). Bold values represent statistically significant results (p < 0.05).*

#### LFDI Map Area

Map area for LFDI did not differ across mapping sessions regardless of stimulation group (Figure 4D). Estimated marginal means of LFDI map area are reported in Table 2. There were no effects of *time* (*F* = 0.947, *p* = 0.396), *stimulation* (*F* = 0.520, *p* = 0.479), or any interaction between *time* and *stimulation* (*F* = 0.546, *p* = 0.584) on LFDI map area between active forms of stimulation and Sham (Figure 4E). LFDI map areas were comparable at the pre timepoint between stimulation groups (*F* = 1.100, *p* = 0.353). Similarly, there was no effect of *time* (*F* = 2.23, *p* = 0.122), *stimulation type* (*F* = 0.91, *p* = 0.431), or any interaction between *time* and *stimulation type* (*F* = 0.57, *p* = 0.684) on LFDI motor map volume across the three intervention groups (Figure 4F). Fixed parameter effects of LFDI area across both stimulation and stimulation type are reported in Tables 3, 4.

### LFDI Secondary Motor Mapping Outcomes

#### LFDI Centre of Gravity

We investigated whether LFDI COG in the 2D x- or y-plane shifted following motor learning and stimulation between mapping sessions. Estimated marginal means are reported in Table 2. Fixed effects parameter estimates for LFDI COG in the medial-lateral plane (x-axis, COG-x) and anterior-posterior plane (y-axis, COG-y) between stimulation (Active or Sham) and stimulation type (tDCS, HD-tDCS and Sham) are reported in Tables 3, 4.

**FIGURE 5:**
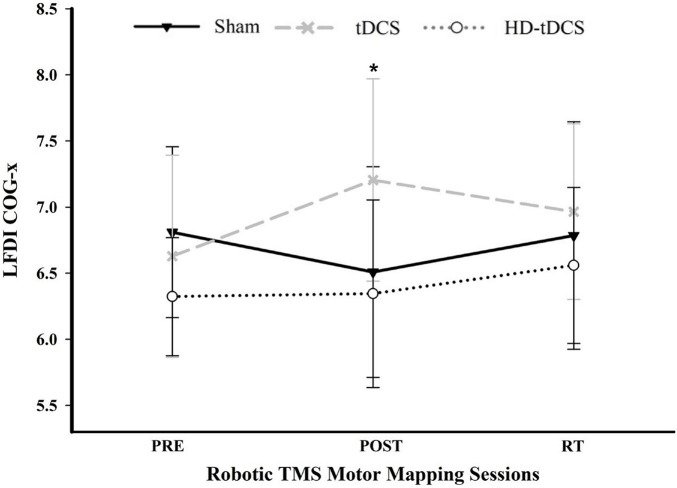
COG-x across mapping sessions. Mean LFDI COG-x (mm) between intervention groups across time points. Change in COG-x significantly differed between tDCS and Sham from the pre-to-post mapping sessions (*p* = 0.003). Black line = Sham; Gray dashed line = tDCS; Gray dotted line = HD-tDCS; LFDI = left-hand first dorsal interosseous muscle; COG-x = centre of gravity medial-lateral plane; RT = six-weeks retention time. Error bars = standard deviation. *p < 0.05.

There was a significant interaction between *time* and *stimulation type* on LFDI COG-x (*F* = 2.83, *p* = 0.038) between intervention groups (Figure 5). From pre-to-post, the degree of change in COG-x differed between tDCS and Sham [*t*_(38)_ = 3.225, *p* = 0.003]. There was no effect of *time* (*F* = 1.32, *p* = 0.278) or *stimulation type* (*F* = 1.27, *p* = 0.305) on LFDI COG-x. LFDI COG-y showed no effects of *time* (*F* = 0.59, *p* = 0.558), *stimulation type* (*F* = 1.46, *p* = 0.257), or any interaction between *time* and *stimulation type* (*F* = 1.45, *p* = 0.236) across intervention groups. Between Active or Sham, LFDI COG-x showed no effect of *time* (*F* = 0.71, *p* = 0.497), *stimulation* (*F* = 0.001, *p* = 0.967) or any interaction between *time* and *stimulation type* (*F* = 3.10, *p* = 0.056). LFDI COG-y also showed no effect of *time* (*F* = 0.59, *p* = 0.562), *stimulation* (*F* = 1.08, *p* = 0.322) or any interaction between *time* and *stimulation type* (*F* = 3.02, *p* = 0.060).

#### LFDI Hotspot Magnitude

Left-Hand First Dorsal Interosseous hotspot magnitude did not differ across mapping sessions regardless of stimulation group. Estimated marginal means for LFDI hotspot magnitude are reported in Table 2. Fixed effects parameter estimates for LFDI hotspot magnitude between stimulation (Active or Sham) and stimulation type are reported in Tables 3, 4.

There were no effects of *time* (*F* = 1.87, *p* = 0.168), *stimulation* (*F* = 1.51, *p* = 0.233), or any interaction between *time* and *stimulation* (*F* = 0.61, *p* = 0.547) on LFDI hotspot magnitude between Active stimulations and Sham. Similarly, there was no effect of *time* (*F* = 1.35, *p* = 0.272), *stimulation type* (*F* = 1.24, *p* = 0.312), or any interaction between *time* and *stimulation type* (*F* = 0.30, *p* = 0.875) on LFDI hotspot magnitude across the three intervention groups.

### Motor Map Outcomes for Left-Hand Secondary Muscles and Right-Hand Muscles

Map outcomes for left-hand secondary muscles (APB and ADM) and right-hand muscles are summarised in Supplementary Table 1. In secondary muscles of the trained left-hand, we observed significant interactions for LAPB and LADM COG-x between *time* and *stimulation* (LAPB *F* = 6.62, *p* = 0.003; LADM *F* = 7.40, *p* = 0.002) and *stimulation type* (LAPB *F* = 4.23, *p* = 0.006; LADM *F* = 5.16, *p* = 0.002). From pre-to-RT, change in COG-x differed between Active (tDCS and HD-tDCS) and Sham [LAPB *t*_(38)_ = −3.537, *p* = 0.001; LADM *t*_(38)_ = −3.688, *p* < 0.001). No effects of *stimulation, stimulation type*, or *time* were observed on map volume, area, hotspot magnitude, or COG-y, within the left-hand secondary muscles.

Right-hand first dorsal interosseous map volume and area across time and stimulation type are depicted in Supplementary Figure 1. No significant effects were observed on map volume, area, hotspot magnitude, COG-x, or COG-y in any of the untrained right-hand muscles.

### Purdue Pegboard Task Performance and First Dorsal Interosseous Map Volume and Area

**FIGURE 6:**
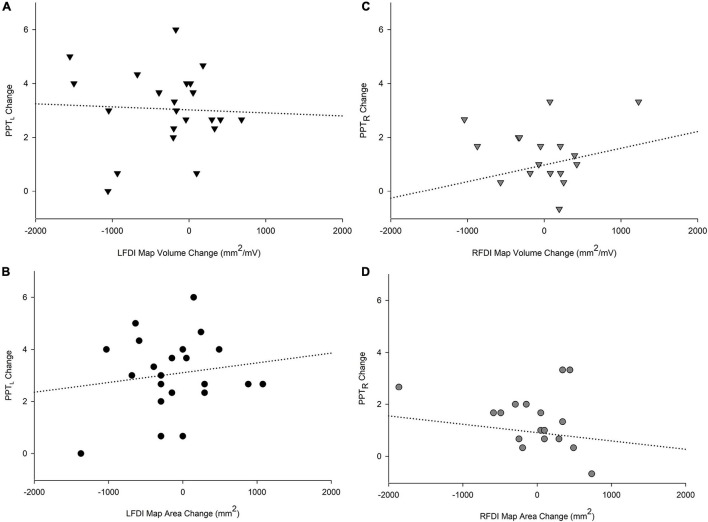
Relationships of PPT motor performance with map volume (mm^2^/mV) and area (mm^2^). Change in PPT_*L*_ scores with LFDI map volume **(A)** and area **(B)**. Change in PPT_*R*_ scores with RFDI map volume **(C)** and area **(D)**. Linear regression showed no significant relationship between change in FDI map volume and area with change in PPT scores between the pre and post in either hand. LFDI = left-hand first dorsal interosseous muscle; RFDI = right-hand first dorsal interosseous muscle; PPT = change in number of pegs from pre-to-post; PPT_*L*_ = Left-hand; PPT_*R*_ = Right-hand.

We investigated associations between the change in motor performance scores of the left-hand (PPT_L_) and right-hand (PPT_R_) and the change in FDI map volume and area from the pre-to-post mapping sessions (Figure 6). In the trained left-hand, linear regression found no relationship between change in PPT_L_ performance and change in LFDI map volume (*R* = 0.196, *t* = −0.451, *p* = 0.657) or area (*R* = 0.204, *t* = 0.516, *p* = 0.612) (Figures 6A,B). Similarly in the untrained right-hand, no relationship between the change in PPT_R_ performance and the change in RFDI map volume (*R* = 0.281, *t* = 1.062, *p* = 0.303) or area (*R* = 0.158, *t* = −0.351, *p* = 0.730) was found (Figures 6C,D). Lastly, linear regression found no significant relationship between change in map volume or area of the left- and right-hand secondary muscles (APB and ADM) and change in PPT from pre-to-post mapping sessions.

### Safety and Tolerability

Robotic TMS motor mapping was well tolerated by all participants. In 65 motor mapping sessions, no serious adverse events occurred. All reported events were minor (graded as mild or moderate) and decreased over time. The most common adverse event, reported as total number of occurrences, were neck-pain (*n* = 18, 25%) and headache (*n* = 16, 22%) Other events included unpleasant tingling (*n* = 7, 10%), light headedness (*n* = 5, 7%), and nausea (*n* = 1, 1%). On average, participants ranked their TMS session 4th, between a “birthday party” and “long car ride”, compared to other daily activities. tDCS safety and tolerability was also favourable and is reported elsewhere (Cole et al., 2018).

## Discussion

We investigated the effects of natural and modulated (tDCS and HD-tDCS) motor learning on robotic TMS motor maps. There were no significant effects of time or group on FDI motor map volume, area, or hotspot magnitude of the left-hand (trained) or right-hand (untrained), or within any of the secondary muscles. We did observe interactions between time and group on COG-x in muscles of the left-hand over time. Map metrics did not correlate with behavioural changes. Our results suggest robotic TMS motor mapping is a potentially valuable tool for studying plasticity in the developing motor system, but multiple factors must be considered, most notably the large variability in maps across individuals and time (Ngomo et al., 2012).

Primary motor cortex plays a pivotal role during skilled voluntary movements and learning of motor skills (Cohen et al., 1993; Rossi et al., 1999; Sanes and Donoghue, 2000; Muellbacher et al., 2001; Rioult-Pedotti and Donoghue, 2003). TMS has advanced our knowledge of such M1 plasticity, revealing changes in corticospinal excitability and cortical reorganization in response to upper-limb behavioural training (Pascual-Leone et al., 1995b, a; Classen et al., 1998; Butefisch et al., 2000; Ziemann et al., 2001; Gallasch et al., 2009). However, how motor mapping can further inform these mechanisms remains poorly understood. Robotic TMS may offer additional potential in the study of M1 neurophysiology and plasticity and may be particularly applicable in pediatric populations for its improved accuracy, movement-compensation, and increasingly well-established safety and tolerability profile (Zewdie et al., 2020). We recently demonstrated that robotic TMS can safely quantify M1 neurophysiology in healthy children (Grab et al., 2018) and investigated motor map outcomes and their associations with motor performance (Giuffre et al., 2021).

The present study is the first to demonstrate the application of such robotic TMS motor map outcomes to explore the underlying M1-plasticity effects of multiple days of modulated learning in healthy children (Cole et al., 2018). Our findings suggest COG may be a valuable neurophysiological motor mapping outcome. Following five consecutive days of intervention, the weighted cortical motor map of the trained LFDI appeared to shift differently between those participants receiving active tDCS compared to Sham. The significance of this finding is supported by our reliability study of robotic TMS motor mapping measures over both short- and long-term timeframes in young adults (Giuffre et al., 2020). Our reported standard error of COG-x (0.365) was below our previously reported standard error of measurement for COG-x (0.40). Such observations of COG-x being more reliable than other map outcomes is consistent with other reports (Wassermann et al., 1992; Mortifee et al., 1994; Jones–Lush et al., 2010; Ngomo et al., 2012; Littmann et al., 2013). COG may reflect changes in cortical physiology of upper-limb muscles following behavioural training or tDCS-modulated motor skill learning. Medial-lateral shifts in COG have been previously reported to shift medially with age in preadolescents (Säisänen et al., 2021) following one-hour of motor training (Liepert et al., 1999; Tegenthoff et al., 2004) and suggested to reflect shifts in motor cortex excitability (Wassermann, 2002) and reorganization following motor learning (Liepert et al., 2000). As M1 is distributed in a medial-lateral plane, shifts along COG-x potentially identify scalp coordinates corresponding to a larger number of the most excitable corticospinal neurons influencing recorded muscle activity (Röricht et al., 2001). Other reports suggest COG may be a more precise measure of anatomical reorganization assessable by TMS, although additional mapping outcomes such as map volume, area, and hotspot magnitude are more commonly investigated.

Accordingly, our primary map outcome measures were area, volume, and hotspot magnitude. We originally hypothesised that map volume and area would increase following learning and stimulation, based in part on evidence of increased cortical excitability (MEP amplitude) following anodal tDCS in healthy adults and children (Nitsche and Paulus, 2000; Nitsche et al., 2003; Ciechanski and Kirton, 2017; Cole et al., 2018) and enlarged motor map representations following motor performance alone in both animal (Nudo et al., 1996) and human models (Pascual-Leone et al., 1995a). Since map volume quantifies MEP amplitude at responsive grid-points, taking map area into account, we predicted the effects of tDCS stimulation might unveil different patterns of change within these M1 representations of hand muscles. Both measures have the potential to be complementary metrics capable of quantifying M1-plasticity effects such as strengthened synaptic connections and unmasking of latent horizontal connections (Huntley, 1997; Sanes and Donoghue, 2000). Contrary to our hypothesis, however, map volume, area, and cortical excitability showed no significant effect of time or group. Since executing the current study, we have learned that inter-session reliability of these outcomes is moderate with large minimal detectable differences estimated at more than 40–50% (Giuffre et al., 2020). Accordingly, with our modest sample divided into three treatment groups, we were likely underpowered to show significant changes in these standard map metrics. Additional reasons behind the absence of findings may stem from diverse individual variability in tDCS-induced changes in excitability, individual differences in motor skill learning, or differences in tDCS current density among participants (Ciechanski et al., 2018).

Increased map volume and area have been associated with repeated motor task learning (Pearce et al., 2000) and acquiring motor skills (Pascual-Leone et al., 1995b; Reis and Fritsch, 2011). In healthy subjects, MEP amplitude and cortical representations of fingers (map size) increased following repetitive practice of a piano sequence (Pascual-Leone et al., 1995a), while showing no change following repeated PPT performance (Garry et al., 2004) or during non-specific motor training (Ngomo et al., 2012). It is postulated that while behaviour improves, cortical output maps to the muscles involved become progressively larger until explicit knowledge is achieved, after which they may reduce in size (Pascual-Leone et al., 1994). For instance, while many TMS studies reported behavioural training increases cortical excitability (MEP amplitude), suggesting a correlation with functional outcome (Pascual-Leone et al., 1995a; Muellbacher et al., 2001; Ziemann et al., 2001; Garry et al., 2004), others have reported no change (Carroll et al., 2002; Ngomo et al., 2012). Inconsistent findings are also found in motor map outcomes, such as volume and area, following behavioural training though these have been studied almost exclusively in adults (Brasil-Neto et al., 1992; Wilson et al., 1993; Mortifee et al., 1994; Thickbroom et al., 1998; Malcolm et al., 2006). These may represent potential reasons for our results showing no definitive changes in motor map volume and area following motor training. That stimulation with tDCS or HD-tDCS might also alter the natural direction of effects of motor learning on motor maps must also be considered.

More expansive representations of a trained sequence of movements in M1 have been associated with performance, suggesting specific representations of movement sequences may be implemented at the cortical level as a new functional unit and distributions of cortical sites may be functionally linked during coordinated movements (Tyč and Boyadjian, 2011). As we demonstrated in the behavioural arm of the interventional trial on which the current study is based, days 4 and 5 showed the most significant increases of tDCS-induced effects on motor performance (Cole et al., 2018). We observed similar results in an earlier trial of tDCS-enhanced motor learning in children (Ciechanski and Kirton, 2017). The effect of time (day of training) appears to be dynamic and possibly altered by stimulation, adding yet another source of variance between individuals to our measurements which were limited to 3 timepoints. Acquiring TMS mapping measures on each day of such interventional trials could shed light on such shorter-term, day-to-day changes in cortical representations (map volume or area) during the course of stimulation-induced motor learning but may be limited by resource utilization and participant compliance.

To date, there is no gold standard for TMS motor mapping that provides the highest accuracy of measurements. Mäki and Ilmoniemi (2010) showed variability of MEP amplitudes at the same cortical grid-point, one-third of the largest responses were on average 10 times higher than one-third of the lowest responses (Mäki and Ilmoniemi, 2010). Estimating the size of motor representations based on MEP amplitudes obtained within a finite number of stimulation points is challenging (Julkunen, 2014) and depends on the accuracy of stimulation, density of stimulus locations, and MEP variability at the borders of the maps (Brasil-Neto et al., 1992; Classen et al., 1998; Chernyavskiy et al., 2019). Other groups have used a greater number of stimuli (6–10 pulses) per grid-point to accommodate (Cirillo et al., 2010; Ngomo et al., 2012) and reduce variability (Bastani and Jaberzadeh, 2012; van de Ruit et al., 2015), however, the feasibility of this approach in the developing brain is questionable. Additional recent investigations using neuronavigated single biphasic TMS pulses suggest increased efficacy to induce motor responses (Pitkänen et al., 2018), which may accommodate motor mapping challenges in young children, such as high motor thresholds (Julkunen, 2014; van de Ruit et al., 2015), though investigations in pediatric populations are lacking. Robotic neuronavigated TMS may be a useful tool to overcome these and other challenges in children. In addition to reducing human error of accurate coil placement, neuronavigated robotic TMS reduces acquisition time, provides consistent coil positioning, and near real-time motion correction, accommodating subject movement (Ginhoux et al., 2013). Robotic motor mapping appears safe, feasible in children (Grab et al., 2018; Giuffre et al., 2021), and is well-supported by growing evidence of TMS safety in children (Zewdie et al., 2020).

An ability to measure motor map plasticity has translational implications for children with early brain injury and cerebral palsy, the leading cause of lifelong neurological disability. The sensorimotor network is one of the earliest developing networks, well-established very early in life but highly refined throughout of development where motor maps may undergo dramatic developmental plasticity (Khazipov and Milh, 2018). How these natural processes are altered after early unilateral injury such as perinatal stroke are increasingly understood from preclinical models (Martin et al., 2007; Wen et al., 2018) and human imaging and brain mapping studies (Kirton et al., 2021). A recent mouse model of perinatal stroke evaluated motor map size and movement latency following cortical stimulation and motor training (Zhang et al., 2021). In line with our findings, motor map area (size) showed no overall expansion following 10 min a day over three-weeks of pellet training. Interestingly, mice with smaller map areas at baseline showed the greatest improvement in skilled forelimb training. TMS motor maps appear to be altered in children with unilateral cerebral palsy (CP) undergoing therapeutic interventions. For example, Friel et al. (2016) has shown increased motor map area (size) and MEP amplitudes of the affected hand in children with unilateral spastic CP following 3-weeks (6 h/day, 5 days/week) of bimanual therapy (Friel et al., 2016). Additional investigations by their group have shown medial-lateral shifts in FDI COG, in addition to investigating size and excitability of cortical motor maps, may lead toward further defining the relationship between changes in motor maps and motor function in children with CP (Kesar et al., 2012; Marneweck et al., 2018). Such models are informing large-scale randomised neuromodulation clinical trials (Kirton et al., 2016, 2017) where the addition of robotic TMS mapping with comprehensive outcomes such as COG before and after modulation may shed further light on mechanisms of interventional plasticity.

In addition to the above limitations, additional challenges are acknowledged. Our study could not control for other factors that may influence map size, such as the influence of hand use throughout the intervention (Ngomo et al., 2012). For instance, musicians and racket ballplayers (Pearce et al., 2000) who utilise their hand muscles more than the average person display different neurophysiology and organization of cortical motor networks. Our study was based on a secondary aim of the primary interventional tDCS trial, powered to determine the behavioural effects of tDCS on motor learning in children. Combined with new knowledge of TMS mapping reliability (Giuffre et al., 2020), it is clear that we had very modest power to detect true changes in map parameters. This emphasises the need for careful power calculations in future motor mapping studies. Additional limitations are due to the potential differences in electric field strength and distribution in our pediatric sample. Advances in computer finite element modeling (FEM) and realistic 3D head models have enabled tDCS-induced current to be predicted and modeled through the cortex (Miranda et al., 2006; Wagner et al., 2006; Bikson et al., 2012). The development of high-resolution derived head models allows for more accurate and precise modeling of current, though special considerations are required when applying tDCS in children. An early current modeling case study suggested tDCS-induced electric fields may be stronger in children (Kessler et al., 2013). Supplementing these findings in the largest pediatric sample to date, our group recently demonstrated children incur increased current densities and distributions compared to adolescents and adults (Ciechanski et al., 2018). These differences may be attributed to age-related differences in developmental changes within grey and white matter and skull thickness. As children have thinner skulls, stronger electric fields may be induced, resulting in more expansive current in underlying tissue (Opitz et al., 2015; Ciechanski et al., 2018). Recently, HD-tDCS was proposed to offer more focal current delivery and optimised targeting (Datta et al., 2009), however, investigations of modeling HD-tDCS current distributions in pediatrics are lacking. In a preliminary investigation, we studied potential differences in electric field modeling of conventional tDCS and HD-tDCS in the pediatric sample described in this study. We observed lower peak electric fields in HD-tDCS compared to tDCS and found no significant associations between electric fields and motor performance of the two stimulation groups (Giuffre et al., 2019a). As a larger sample of participants is needed to determine differences in electric field distribution of tDCS and HD-tDCS, we cannot account for possible differential effects of tDCS on motor maps. Additional investigations of current modeling various tDCS montages, especially in pediatric and clinical populations, are needed to optimise tDCS enhancement of motor function and advance therapies for clinical populations.

Patterns of motor map volume and area within participants revealed large variability that likely limited our ability to detect differences. Variability can be attributed to both subject factors (age, genetics, and sleep) (Antal et al., 2010; Li Voti et al., 2011; Ngomo et al., 2012), as well as measurement error. Variable subthreshold activation of corticospinal outputs at rest, background EMG activity, and focality of TMS-induced electric currents are all considered and can only partially be controlled. These factors may have contributed to our findings in secondary hand muscles. As different muscle representations display unique pyramidal and interneuronal orientations, the TMS coil may differentially active unique muscle representations. Although many horizontally oriented neuronal elements are perpendicular to the central sulcus, the depth from the scalp at which these elements initiate MEP response to TMS are difficult to evaluate and likely differs in children. An additional limitation may relate to our use of the LFDI RMT to determine the mapping threshold across all muscles. Although this may provide relative cortical representations of additional hand muscles and is highly practical, it cannot account for differences in thresholds, orientation, and other specific factors unique to each muscle representation that might only be determined with each mapped individually.

## Conclusion

In summary, robotic TMS motor mapping is feasible and well-tolerated in children. The maps generated can estimate neurophysiological measures relevant to M1 physiology and plasticity. Large effects on traditional mapping outcomes such as area and volume were not observed but shifts in COG may represent an informative measure. Fully powered TMS motor mapping investigations are needed in the developing brain to determine utility in understanding mechanisms of motor developmental and interventional plasticity.

## Data Availability Statement

The original contributions presented in the study are included in the article/Supplementary Material, further inquiries can be directed to the corresponding author.

## Ethics Statement

The studies involving human participants were reviewed and approved by University of Calgary Research Ethics Board (REB16-2474). Written informed consent and assent when applicable to participate in this study was provided by the participants’ legal guardian/next of kin.

## Author Contributions

AG: conceptualization, methodology, formal analysis, ethics, recruiting, investigation, data collection, supervision, and writing—original draft. EZ, JW, and LC: conceptualization, methodology, formal analysis, ethics, recruiting, investigation, data collection, and writing—manuscript and editing. HC: conceptualization, methodology, formal analysis, investigation, data collection, and writing—manuscript and editing. H-CK: conceptualization, methodology, formal analysis, recruiting, investigation, data collection, and writing—manuscript and editing. AB: formal analysis, investigation, and writing—manuscript and editing. AK: conceptualization, methodology, ethics, investigation, writing—original draft, and supervision. All authors contributed to the article and approved the submitted version.

## Conflict of Interest

The authors declare that the research was conducted in the absence of any commercial or financial relationships that could be construed as a potential conflict of interest.

## Publisher’s Note

All claims expressed in this article are solely those of the authors and do not necessarily represent those of their affiliated organizations, or those of the publisher, the editors and the reviewers. Any product that may be evaluated in this article, or claim that may be made by its manufacturer, is not guaranteed or endorsed by the publisher.

## Footnotes

1
https://www.jamovi.org
