# Supplementary material for: Effects of Transcranial Direct Current Stimulation and High-Definition Transcranial Direct Current Stimulation Enhanced Motor Learning on Robotic Transcranial Magnetic Stimulation Motor Maps in Children
Source: Front Hum Neurosci. 2021 Oct 6;15:747840. doi: 10.3389/fnhum.2021.747840 (PMC8526891; doi:10.3389/fnhum.2021.747840)
Supplement: Supplementary file 1 [file Data_Sheet_1.PDF]

Supplementary Figure 1. RFDI map volume and area across robotic TMS motor mapping sessions. (A) RFDI map volume (mm^2/mV) of each participant across mapping sessions. **(B)** RFDI map volume (mm^2/mV) in active stimulation groups (tDCS and HD-tDCS) and Sham. **(C)** RFDI map volume (mm^2/mV) in intervention groups (tDCS, HD-tDCS and Sham). **(D)** RFDI map area (mm^2) of each participant across motor mapping sessions. **(E)** RFDI map area (mm^2) in active stimulation groups (tDCS and HD-tDCS) and Sham. **(F)** RFDI map area (mm^2) in intervention groups (tDCS, HD-tDCS and Sham). *Map volume (mm^2/mV) = Cumulative active map area * MEP amplitude (mV) at each responsive grid-point; Map area (mm^2) = Grid spacing ($49mm^2$) * number of responsive grid-points; Black line with triangle = Sham participants; Gray dashed line with X = tDCS participants; Gray dotted line with circle = HD-tDCS; RFDI = right-hand first dorsal interosseous muscle; Pre = baseline; Post = day 5; RT = six-weeks retention time. Box ends are interquartile range, the line is median, and the whiskers are 5-95%.*

Supplementary Table 1. Estimated marginal means of motor map outcomes in secondary left- and right-hand muscles. *L = left-hand; R = right-hand. FDI = left-hand first dorsal interosseous muscle; APB = left-hand abductor pollicus brevis; ADM = abductor digit minimi; Hotspot magnitude = MEP amplitude(mV); COG = centre of gravity, COG-x (medial-lateral plane), COG-y (anterior-posterior plane); Pre = baseline; Post = day 5; RT = six-weeks retention time.*

Estimated Marginal Means Intervention Groups (tDCS, HD-tDCS, Sham)

		Time			Group			Time * Group								
								tDCS			HD-tDCS			Sham		
	Muscle	PRE	POST	RT	tDCS	HD-tDCS	Sham	PRE	POST	RT	PRE	POST	RT	PRE	POST	RT
**Volume (mm^2/mV)**	LAPB	425 (80.1)	359 (80.1)	294 (80.1)	320 (115)	220 (123)	537 (123)	383 (133)	315 (133)	263 (133)	272 (142)	207 (142)	181 (142)	620 (142)	555 (142)	436 (142)
	LADM	167 (26.1)	119 (26.1)	143 (26.1)	184 (32.8)	102 (35.0)	142 (35.0)	211.1 (43.2)	163.9 (43.2)	176.6 (43.2)	112.9 (46.2)	90.2 (46.2)	104 (46.2)	175.5 (46.2)	101.9 (46.2)	149.5 (46.2)
	RFDI	821 (159)	789 (159)	630 (162)	623 (194)	812 (243)	806 (206)	750 (247)	391 (247)	728 (260)	891 (313)	1078 (313)	466 (313)	823 (264)	897 (264)	697 (264)
	RAPB	390 (168)	455 (168)	481 (171)	477 (164)	334 (204)	515 (172)	286 (260)	279 (260)	866 (278)	345 (329)	462 (329)	196 (329)	540 (278)	625 (278)	380 (278)
	RADM	224 (104)	325 (104)	188 (105)	336 (109)	198 (135)	204 (114)	242 (160)	439 (160)	328 (171)	229 (203)	244 (203)	121 (203)	203 (172)	294 (172)	115 (172)
**Area (mm^2)**	LAPB	1036 (104)	959 (104)	922 (104)	1009 (139)	828 (149)	1080 (149)	1084 (172)	1023 (172)	919 (172)	882 (184)	889 (184)	714 (184)	1141 (184)	966 (184)	1134 (184)
	LADM	827 (90.4)	717 (90.4)	715 (90.4)	813 (106)	695 (113)	761 (113)	772 (150)	815 (150)	851 (150)	805 (160)	686 (160)	595 (160)	903 (160)	651 (160)	728 (160)
	RFDI	1123 (103)	1078 (103)	1018 (105)	1140 (128)	947 (161)	1132 (136)	1280 (160)	937 (160)	1201 (169)	1019 (203)	1078 (203)	745 (203)	1071 (171)	1218 (171)	1106 (171)
	RAPB	940 (119)	1071 (119)	996 (121)	1015 (133)	921 (166)	1071 (141)	956 (184)	1011 (184)	1079 (195)	862 (233)	1000 (233)	902 (233)	1001 (197)	1204 (197)	1008 (197)
	RADM	881 (141)	982 (141)	756 (142)	915 (182)	882 (228)	821 (193)	882 (219)	1011 (219)	852 (229)	941 (277)	941 (277)	764 (277)	819 (234)	994 (234)	651 (234)
**Hotspot magnitude (mV)**	LAPB	1.142 (0.165)	0.856 (0.165)	0.741 (0.165)	0.800 (0.236)	0.592 (0.252)	1.346 (0.252)	1.016 (0.272)	0.791 (0.272)	0.594 (0.272)	0.657 (0.291)	0.534 (0.291)	0.584 (0.291)	1.751 (0.291)	1.241 (0.291)	1.044 (0.291)
	LADM	0.408 (0.054)	0.329 (0.054)	0.379 (0.054)	4.66 (0.077)	0.283 (0.077)	3.67 (0.077)	0.504 (0.090)	0.412 (0.090)	0.483 (0.090)	0.324 (0.096)	0.249 (0.096)	0.276 (0.096)	0.396 (0.096)	0.327 (0.096)	0.379 (0.096)
	RFDI	2.03 (0.341)	1.67 (0.341)	1.65 (0.341)	1.87 (0.508)	1.14 (0.543)	2.34 (0.543)	2.02 (0.564)	1.81 (0.564)	1.79 (0.564)	1.25 (0.603)	1.15 (0.603)	1.02 (0.603)	2.80 (0.603)	2.06 (0.603)	2.15 (0.603)
	RAPB	1.083 (0.216)	1.028 (0.216)	0.920 (0.219)	0.822 (0.273)	0.750 (0.342)	1.460 (0.289)	0.765 (0.335)	0.676 (0.335)	1.024 (0.351)	0.836 (0.423)	0.852 (0.423)	0.562 (0.423)	1.650 (0.358)	1.557 (0.358)	1.173 (0.358)
	RADM	0.521 (0.109)	0.577 (0.109)	0.391 (0.110)	0.563 (0.14)	0.425 (0.176)	0.501 (0.149)	0.639 (0.169)	0.620 (0.169)	0.430 (0.177)	0.444 (0.214)	0.472 (0.214)	0.360 (0.214)	0.481 (0.181)	0.639 (0.181)	0.383 (0.181)
**COG-x (mm)**	LAPB	0.152 (0.107)	0.166 (0.107)	0.014 (0.107)	0.204 (0.116)	(0.218) (0.124)	-0.090 (0.124)	0.554 (0.178)	0.307 (0.178)	-0.247 (0.178)	0.256 (0.190)	0.327 (0.190)	0.071 (0.190)	-0.354 (0.190)	-0.135 (0.190)	0.218 (0.190)
	LADM	0.118 (0.113)	0.213 (0.113)	0.095 (0.113)	0.215 (0.138)	0.320 (0.148)	-0.108 (0.148)	0.524 (0.188)	0.322 (0.188)	-0.202 (0.188)	0.254 (0.201)	0.479 (0.201)	0.226 (0.201)	-0.423 (0.201)	-0.162 (0.201)	0.261 (0.201)
	RFDI	0.092 (0.251)	-0.003 (0.260)	-0.140 (0.260)	-0.361 (0.296)	0.320 (0.358)	-0.034 (0.317)	0.172 (0.404)	-0.542 (0.404)	-0.714 (0.404)	0.318 (0.467)	0.512 (0.507)	0.130 (0.507)	-0.215 (0.432)	-0.051 (0.432)	0.165 (0.432)
	RAPB	0.026 (0.239)	-0.044 (0.246)	-0.084 (0.246)	-0.367 (0.308)	0.275 (0.368)	-0.011 (0.329)	-0.063 (0.385)	-0.551 (0.385)	-0.487 (0.385)	0.219 (0.444)	0.435 (0.478)	0.172 (0.478)	-0.079 (0.411)	-0.016 (0.411)	0.063 (0.411)
	RADM	0.005 (0.230)	-0.054 (0.237)	-0.071 (0.237)	-0.350 (0.295)	0.254 (0.353)	-0.025 (0.316)	-0.007 (0.370)	-0.524 (0.370)	-0.517 (0.370)	0.179 (0.427)	0.399 (0.460)	0.184 (0.460)	-0.157 (0.396)	-0.037 (0.396)	0.120 (0.396)
**COG-y (mm)**	LAPB	0.081 (0.138)	0.153 (0.138)	0.072 (0.138)	0.267 (0.146)	0.099 (0.156)	-0.060 (0.156)	0.146 (0.229)	0.400 (0.229)	0.254 (0.229)	-0.040 (0.244)	0.149 (0.244)	0.189 (0.244)	0.135 (0.244)	-0.090 (0.244)	-0.225 (0.244)
	LADM	0.172 (0.148)	0.084 (0.148)	-0.087 (0.148)	0.280 (0.158)	0.049 (0.169)	-0.160 (0.169)	0.243 (0.245)	0.420 (0.245)	0.176 (0.245)	0.067 (0.262)	0.073 (0.262)	0.006 (0.262)	0.204 (0.262)	-0.240 (0.262)	-0.444 (0.262)
	RFDI	-0.162 (0.250)	-0.190 (0.258)	-0.013 (0.258)	-0.466 (0.311)	-0.085 (0.373)	0.187 (0.332)	-0.098 (0.402)	-0.699 (0.402)	-0.601 (0.402)	-0.246 (0.464)	-0.152 (0.502)	0.143 (0.502)	-0.141 (0.430)	0.280 (0.430)	0.420 (0.430)
	RAPB	-0.114 (0.260)	-0.185 (0.269)	-0.060 (0.269)	-0.496 (0.313)	-0.049 (0.376)	0.186 (0.334)	-0.157 (0.418)	-0.743 (0.418)	-0.586 (0.418)	-0.166 (0.418)	-0.090 (0.524)	0.109 (0.524)	-0.019 (0.447)	0.278 (0.447)	0.298 (0.447)
	RADM	-0.115 (0.258)	-0.205 (0.266)	-0.076 (0.266)	-0.520 (0.317)	-0.128 (0.381)	0.252 (0.339)	-0.058 (0.415)	-0.780 (0.415)	-0.722 (0.415)	-0.225 (0.479)	-0.214 (0.519)	0.056 (0.519)	-0.061 (0.444)	0.378 (0.444)	0.439 (0.444)
