# Supplementary figures and images for: Effects of Transcranial Direct Current Stimulation and High-Definition Transcranial Direct Current Stimulation Enhanced Motor Learning on Robotic Transcranial Magnetic Stimulation Motor Maps in Children
Source: Front Hum Neurosci. 2021 Oct 6;15:747840. doi: 10.3389/fnhum.2021.747840 (PMC8526891; doi:10.3389/fnhum.2021.747840)

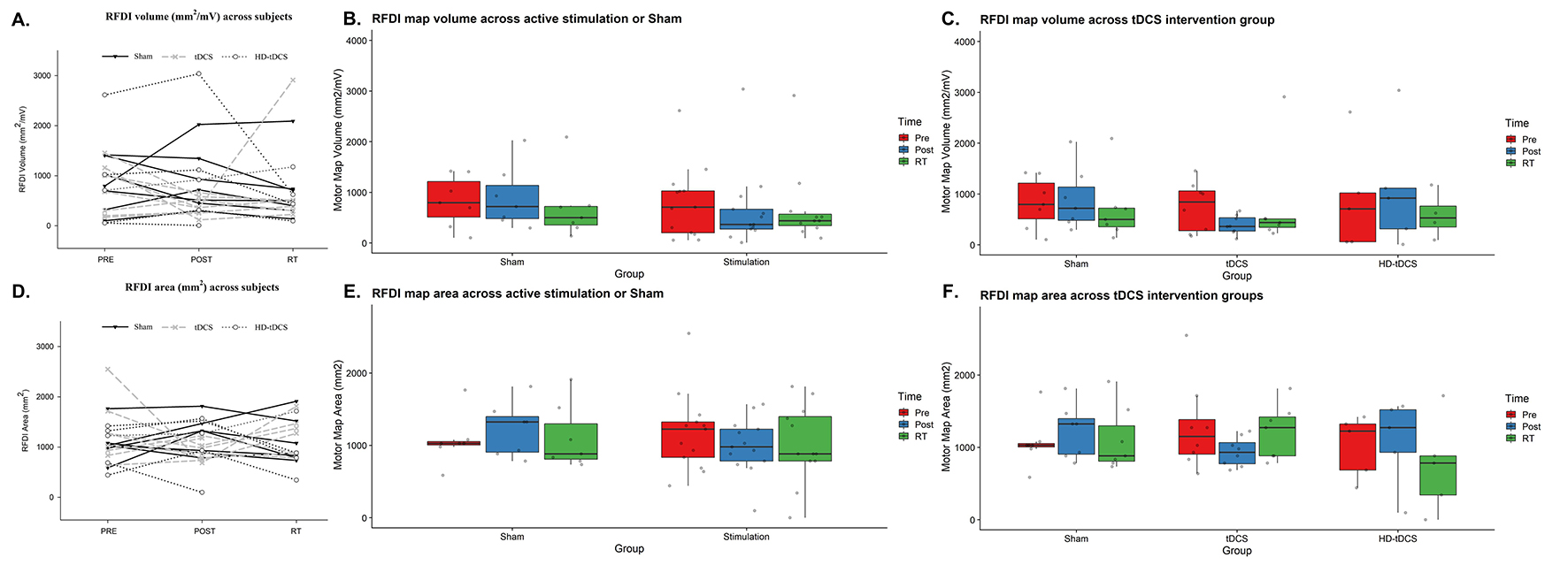

Supplement: Supplementary file 2 [file Image_1.JPEG]
